# Sounds of creativity: musical, creative, and language factors associated with singing and creative singing

**DOI:** 10.1007/s00426-026-02346-x

**Published:** 2026-07-06

**Authors:** Christine Groß, Markus Christiner

**Affiliations:** 1 Jazeps Vitols Latvian Academy of Music, K. Barona Street 1, Riga, LV-1050 Latvia; 2 School of Health, Education, and Social Sciences, SRH University Heidelberg, Ludwig- Guttmann-Straße 6, Heidelberg, 69123 Germany; 3 Department of Child and Adolescent Psychiatry, Saarland University, Homburg, Germany; 4 Music Psychology and Brain Research Section, Department of Psychology, University of Graz, Glacisstraße 27, Graz, 8010 Austria

## Abstract

Singing integrates musical, linguistic, and creative abilities. This study examined how musical aptitude, linguistic skills, and domain-general creativity jointly predict both standard and creative forms of singing. One hundred and eleven adults completed a standardized battery assessing beat perception (CA-BAT), melodic discrimination (MDT), mistuning perception (MPT), musical self-assessment scales, musical engagement, musical socialisation, and creative task-solving competence. Linguistic expressivity was measured through expressive reading of the fable *The North Wind and the Sun*, and linguistic adaptability through spontaneous speech imitation in Tagalog and Mandarin, both rated by native speakers. Participants performed two singing tasks—standard and creative renditions of *Happy Birthday*—evaluated by expert musicians for accuracy and creativity. Stepwise multiple regressions revealed two distinct predictor constellations. Standard singing was best explained by musical engagement, expressive reading, and composite musical ability. Creative singing, by contrast, was predicted by spontaneous speech imitation, expressive reading, and creative problem-solving competence when musical predictors were excluded. These findings indicate that vocal creativity relies on flexible auditory–motor coupling, prosodic expressivity, and cognitive flexibility, rather than musical skill alone. The results highlight the integrative nature of singing as a multimodal form of vocal creativity emerging from interactions among musical, linguistic, and cognitive systems.

**Supplementary Information:**

The online version contains supplementary material available at https://doi.org/10.1007/s00426-026-02346-x.

## Introduction

Singing is a complex vocal behavior present in all human cultures (Hutchins & Peretz, 2012). Its universality has made it an increasingly valuable subject in interdisciplinary research, particularly in studies of the relationship between music and language. A central reason is that singing draws on musical and linguistic abilities, thereby bridging two domains often examined in isolation. Recent research at this intersection has shown consistent transfer from singing to linguistic performance, especially in oral tasks such as pronunciation accuracy, prosodic sensitivity, and verbal memory (Ludke et al., 2014; Snow & Balog, 2002). Across developmental stages, both children and adults learn and retain new lexical items more effectively when words are sung rather than spoken, highlighting the facilitative role of melody and rhythm in language acquisition. Further work indicates that singing ability, most commonly measured with standard singing tasks—that is, tasks requiring the repetition of fixed melodies— is among the strongest predictors of individual differences in foreign language pronunciation, accent mimicry and expressive reading ability (Christiner & Reiterer, 2013; Christiner & Reiterer, 2015, 2018; Christiner et al., 2026). Two main conclusions emerge: (1) melodic structure facilitates verbal memorization, and (2) speech and song share vocal–motor and sensorimotor mechanisms across vocal behaviors (Christiner et al. 2022a, b, c).

When examining the existing body of research on the relationship between singing and language, two main observations emerge. First, studies have predominantly investigated the direction of influence from musicality to language—that is, how singing ability predicts linguistic variables such as pronunciation accuracy or prosodic ability (Christiner & Reiterer, 2013; Christiner & Reiterer, 2015, 2018). While musical training demonstrably enhances (phonological) auditory discrimination, phonological processing, and prosodic perception (Christiner & Groß, 2025; Tierney & Kraus, 2013), research has largely focused on unidirectional transfer from music to language, while transfer from language to music remains less common. It is therefore striking that the reverse relationship—how linguistic ability might predict singing proficiency—has received comparatively little empirical attention (Christiner et al. 2022a, b, c). Considering that speaking, whether in one’s native or a foreign language, occurs far more frequently than singing, this research asymmetry is noteworthy.

Second, one crucial aspect is often overlooked in this interdisciplinary field: the role of spontaneity and creativity. Many experimental paradigms in both musical and linguistic research require participants to perform spontaneous or semi-improvised tasks that rely on cognitive flexibility, divergent thinking, and expressive adaptability (Berkowitz, 2010; Sawyer & Henriksen, 2023). In most studies on singing and language ability, the dimensions of spontaneity and creativity are rarely foregrounded, yet they may represent essential mediating factors in understanding the mechanisms linking singing and speech—particularly within the broader framework of vocal creativity.

Creativity itself represents a domain in its own right, with established assessment traditions such as the Torrance Tests of Creative Thinking (Kaufman & Sternberg, 2019; Runco & Jaeger, 2012). Creativity is widely regarded as a fundamental component of human cognition, typically defined as the ability to generate ideas or behaviors that are both novel and appropriate (Runco & Jaeger, 2012). Creative problem solving has been conceptualized as a multidimensional construct encompassing flexibility, originality, and adaptive solution generation across contexts (Watts et al., 2017). Developmental studies demonstrate that these general skills relate to domain-specific creative productions while maintaining partial independence (Baer, 2016; Barbot et al., 2016). From a cognitive neuroscience perspective, creativity emerges from dynamic interactions between brain networks supporting both domain-general and domain-specific processes (Beaty et al., 2016).

This dual-pathway view underscores the need to examine whether creative singing aligns with general problem-solving competencies or relies on distinct predictors grounded in musical and linguistic expertise. These theoretical perspectives raise the question of how domain-general creative abilities manifest in specific expressive domains. Creativity can appear in many forms, yet the vocal modality is particularly revealing because it bridges music and language.

Musical creativity has been conceptualized as a dynamic, multidimensional process integrating perceptual, cognitive, motor, and affective components in real time (Schiavio & Benedek, 2020), exemplified by improvisation, which demands rapid flexibility, predictive perception–action coupling, and continuous expressive adaptation (Loui, 2018; Vuust et al., 2022).

Following creativity, improvisation and music research, creative singing can be defined as the spontaneous or flexible generation of musical material and expressive variation during vocal performance. Within this framework, it reflects a dynamic process integrating perceptual, cognitive, motor, and affective components in real time and often manifests in improvisation (Loui, 2018; Schiavio & Benedek, 2020).

In contrast, standard singing focuses on the accurate reproduction of fixed melodic and rhythmic structures, emphasizing vocal–motor precision, pitch accuracy, rhythmic stability, and fidelity to a predefined model rather than the generation of novel sung material. The majority of singing studies – including those examining overlaps between singing and language – have relied largely on standard singing tasks, which involve precise reproduction of fixed melodic and rhythmic structures. Those studies that integrated improvisation and creativity demonstrate that improvisation-based instruction enhances fluency and originality in children (Koutsoupidou & Hargreaves, 2009) and that vocal practice can foster creative performance in adults (Chang, 2023). However, general creativity measures do not reliably predict artistic performance in highly specialized musical activities (Madura, 1996). This suggests that domain-general creativity is associated with musical creativity but does not fully account for creative outcomes in specific musical domains (Kaufman & Sternberg, 2019; Runco & Jaeger, 2012). Empirical research shows that creative singing, particularly in improvisational contexts, draws on both domain-general resources (e.g., divergent thinking, cognitive flexibility, working memory) and domain-specific ones (e.g., fine-grained auditory–motor control, vocal technique, and musical knowledge) (Barbot et al., 2016; Loui, 2018). Therefore, singing occupies a unique position: it requires precise motor control of the voice, continuous auditory feedback, linguistic structuring, and expressive variation—thereby constituting a multimodal model system for investigating creative performance (Loui, 2015; Zatorre et al., 2007).

Creative singing—as a vocal behavior that integrates spontaneity, cognitive flexibility, and expressive variation—offers a direct parallel to language tasks such as expressive reading and speech imitation, both requiring vocal motor creativity. Building on this view, expressive reading and speech imitation (e.g., imitating unfamiliar utterances) represent adjacent forms of creative vocal behavior that share underlying mechanisms with singing. Both require prosodic control, emotional modulation, and fine-grained auditory–motor coordination (Patel, 2007; Zatorre & Baum, 2012). These two linguistic tasks—spontaneous speech imitation and expressive reading—are theoretically expected to predict creative singing because both engage flexible auditory–motor coupling and prosodic fluency, core mechanisms underlying improvisational vocal performance. While speech imitation indexes rapid adaptation to novel phonetic–prosodic patterns, expressive reading reflects the ability to shape timing, rhythm, and intonation expressively within familiar linguistic material.

Speech imitation refers to a task commonly used in language aptitude research to assess how quickly and accurately an individual can reproduce unfamiliar language stimuli (Christiner, 2020; Christiner et al., 2023). This task requires rapid adaptation to novel phonological structures that are often unrelated to one’s native language, demanding a high degree of vocal flexibility, phonetic creativity, and innovative problem solving. When reading fables expressively in one’s native language, speakers draw on well-established phonological and semantic knowledge but must creatively reshape it through timing, intonation, and affect—similar to the expressive transformations observed in creative singing. Together, these forms of creative vocal behavior—singing, expressive reading, and speech imitation—engage overlapping neural systems that support auditory prediction, vocal flexibility, and expressive prosody (Schön et al., 2004). Consequently, we expected that linguistic flexibility and expressive prosody would contribute uniquely to creative singing, even when musical ability is held constant. Recognizing this shared foundation provides the conceptual basis for our experimental design: by systematically comparing these tasks within the same participants, we can identify both the convergent and domain-specific predictors of creative vocal performance. This integrative approach allows us to test whether creative singing relies primarily on general cognitive and auditory–motor mechanisms or whether its unique expressive features emerge from specialized interactions between musical and linguistic systems (Christiner et al. 2022a, b, c; Stadler Elmer 2021; Koutsoupidou and Hargreaves 2009; Chang 2023; Madura 1996; Schiavio and Benedek 2020; Loui 2018; Vuust et al. 2022).

Building on these insights, the present study operationalizes creative singing as an accessible form of vocal improvisation that mobilizes both domain-general and domain-specific resources. By contrasting standard and creative renditions of a familiar melody (“Happy Birthday”), we aimed to identify distinct predictor profiles and to test whether vocal creativity reflects general cognitive and auditory–motor mechanisms or depends on specialized interactions between musical and linguistic systems.

Building on these converging findings, it becomes essential to assess spontaneity, flexibility, and creative problem solving as integral components of performance in both language and music. While previous work has mainly emphasized music-to-language transfer, prosody and rhythm provide bidirectional scaffolds that link expressive reading, speech imitation, and singing (Kühnis et al., 2014; Stadler Elmer, 2021; Tierney & Kraus, 2013; Zuk et al., 2014).

Despite these convergences, creative singing cannot be fully captured by domain-general creativity measures alone. While musical aptitude and engagement are strong predictors, spontaneous linguistic imitation and prosodic fluency may also play independent roles. To disentangle these contributions, both shared and unique predictors should be modeled. Given the intercorrelation between musical and linguistic variables, regression analyses must therefore include and exclude musical predictors to determine the incremental role of language and cognition (Budescu, 1993; Cohen, 2013; Dormann et al., 2013; Johnson, 2000).

The present study therefore examines three vocal tasks that differ in both the familiarity of the auditory material and the type of vocal control required. Standard singing involves the reproduction of a highly familiar melody within a well-known tonal system. Expressive reading similarly relies on familiar linguistic material, but requires speakers to shape prosody and emotional expression for an audience. In contrast, speech imitation involves the reproduction of unfamiliar phonetic patterns from an unknown language, placing stronger demands on auditory–motor flexibility. By examining performance across these tasks, the study aims to clarify how musical ability, linguistic skills, and general cognitive factors contribute to different forms of vocal expression.

We hypothesized that (H1) standard singing performance would be primarily predicted by musical aptitude and engagement, reflecting the role of pitch accuracy and rhythmic coordination in conventional vocal production. Conversely, (H2) creative singing would rely more strongly on linguistic adaptability and prosodic fluency—as indexed by spontaneous speech imitation and expressive reading—with additional contributions from domain-general creative problem-solving ability, particularly when musical predictors are excluded.

Building on this theoretical foundation, the present study contrasts predictors of standard and creative singing within a unified analytical framework. Specifically, it investigates (1) the extent to which musical, linguistic, and cognitive variables explain variance in singing performance; (2) whether creative singing relies on overlapping or distinct predictors relative to standard singing; and (3) how these relationships change when musical predictors are controlled. By systematically comparing these pathways, this study aims to clarify whether vocal creativity reflects a generalizable cognitive capacity or an emergent phenomenon arising from the interaction of musical and linguistic expertise.

## Results

### Descriptive statistiscs

Table 1 presents the means, standard deviations, and observed ranges for all variables included in the analyses. The measures cover musical perception, self-reported musical abilities, engagement and socialisation, creative task-solving and innovation competence, and language-related performance, as well as the two singing outcomes.

**Table 1:** Descriptives

Parameters	Mean (M)	Std. Deviation (SD)	Min	Max
Singing Ability Score	4.92	1.56	1.34	8.19
Creative Singing Ability Score	4.69	1.97	0.88	8.38
Composite Musical Ability Score	0.00	0.90	−2.01	1.79
Musical Engagement Score	−0.01	0.83	−1.02	2.10
Musical Socialisation and Relevance Score	−0.02	0.92	−2.08	1.53
Passive Music Listening Score	4.21	2.71	0.00	9.75
Creative Task-Solving and Innovation Competence	6.25	1.56	2.09	9.36
Fabel Reading	5.40	1.20	2.63	6.51
Spontaneous Speech Ability	4.19	0.81	1.80	8.40

## Correlations

To assess the associations between the two singing performance measures (Singing Ability and Creative Singing Ability) and a comprehensive set of musical, creative, and language-related variables, we conducted Pearson correlation analyses. The variables included objective musical perception tests, self-reported musical ability and melody memory, musical engagement, musical socialisation and relevance, passive music listening, creative task-solving and innovation competence, expressive reading, and spontaneous speech imitation. Pearson correlations among Singing Ability, Creative Singing Ability, and all musical, linguistic, cognitive, and demographic variables are presented in Table 2, respectively.

**Table 2:** Pearson correlations among standard singing, creative singing, and musical, linguistic, and cognitive measures

Variable	Singing Ability Score	Creative Singing Ability Score	Composite Musical Ability Score	Musical Engagement Score	Musical Socialisation and Relevance Score	Passive Music Listening Score	Creative Task-Solving and Innovation Competence	Spontaneous Speech Imitation Ability	Fable Reading	Gender
Singing Ability Score	—									
Creative Singing Ability Score	[r]	—								
Composite Musical Ability Score	0.499**	0.725**	0.358**							
Musical Engagement Score	0.543**	0.530**	0.658**	—						
Musical Socialisation and Relevance Score	0.484**	0.596**	0.755**	0.679**	—					
Passive Music Listening Score	0.052	0.073	0.161	0.206*	0.336**	—				
Creative Task-Solving and Innovation Competence	−0.001	0.304**	0.298**	0.256**	0.332**	0.317**	—			
Spontaneous Speech Imitation Ability	0.229*	0.454**	0.409**	0.215*	0.278**	0.041	0.199*	—		
Fable Reading	0.362**	0.330**	0.310**	0.339**	0.374**	0.197*	0.274**	0.205*	—	
Gender	−0.190*	−0.047	−0.074	0.049	−0.121	0.044	−0.007	−0.030	−0.268**	—

Note. *N* = 111. *p* < 0.05*, *p < 0.01

## Regressions

### Singing ability

After inspection of the correlation matrix, a multiple linear regression analysis was performed. In the regression models, all variables that correlated with the Singing Ability Score (the dependent variable) were entered as independent variables. Variables were retained only if the probability of an F-change was below 0.05; therefore, a stepwise method was applied, with the ordering of predictors determined by statistical criteria.

When musical predictors were included, the stepwise regression revealed that Singing Ability was best explained by a combination of the Musical Engagement Score, Fable reading, and the Composite Musical Ability Score, which together accounted for 35.8% of the variance (Table 3). Musical engagement emerged as the strongest predictor, with additional contributions from expressive reading performance and general musical ability.

**Table 3:** Multiple regression models explaining the variance in Singing Ability Score including musical predictors

Predictor	Partial correlation (pr)	*p*-Value
Step 1: *R* = 0.54, F(1, 109) = 45.50, *p* < 0.001		
Musical Engagement Score	0.54	< 0.001
Step 2: *R* = 0.58, F(1, 108) = 5.78, *p* = 0.018		
Musical Engagement Score	0.47	< 0.001
Fable reading	0.20	0.018
Step 3: *R* = 0.60, F(1, 107) = 4.61, *p* = 0.034		
Musical Engagement Score	0.34	0.002
Fable reading	0.18	0.033
Composite Musical Ability Score	0.22	0.034
Dependent variable: Singing Ability Score		

This complementary model specification is methodologically informative, as strong musical predictors can mask the influence of linguistic variables. Presenting both sets of models therefore provides a more differentiated picture of the relative contributions of musical versus linguistic and cognitive predictors (Johnson, 2000).

When musical predictors were excluded, only Fable reading remained a significant predictor of Singing Ability, explaining 13.1% of the variance (Table 4). This indicates that in the absence of musical factors, expressive oral reading skills still accounted for a meaningful proportion of variance in singing performance.

**Table 4:** Multiple regression model explaining the variance in Singing Ability Score excluding musical predictors

Predictor	Partial correlation (pr)	*p*-Value
Step 1: *R* = 0.36, F(1,109) = 16.48, *p* < 0.001		
Fabel reading	0.36	< 0.001

Dependent variable: Singing Ability Score

## Creative singing ability

Following the correlation analyses, a stepwise multiple regression was applied to determine which variables best accounted for differences in creative Singing Ability. Only predictors showing significant bivariate associations with the dependent measure were considered, and variables were retained in the model when the probability of the F-change was below 0.05. In the first step, the Composite Musical Ability Score emerged as the strongest single predictor, accounting for 52.6% of the variance. The inclusion of Spontaneous Speech Imitation Ability in the second step increased the explained variance to 55.5%. Together, these two factors represent substantial contributors to performance in the creative singing task (Table 5).

**Table 5:** Multiple regression models explaining the variance in Creative Singing Ability Score

Predictor	Partial correlation (pr)	*p*-Value
Step 1: *R* = 0.73, F(1, 109) = 120.80, *p* < 0.001		
Composite Musical Ability Score	0.73	< 0.001
Step 2: *R* = 0.75, F(1, 108) = 7.22, *p* = 0.008		
Composite Musical Ability Score	0.65	< 0.001
Spontaneous Speech Imitation Ability	0.19	0.008
Dependent variable: Creative Singing Ability Score		

When musical predictors were excluded, a different profile emerged. In this model, Spontaneous Speech Imitation Ability was entered first, explaining 20.6% of the variance. The addition of Fable reading in the second step raised the explained variance to 26.5%, and in the third step, Creative Task-Solving and Innovation Competence further increased the explained variance to 29.2% (Table 6). This pattern highlights that, in the absence of musical factors, creative singing ability is significantly shaped by both linguistic expressivity and domain-general creative problem solving skills.

**Table 6:** Multiple regression models explaining the variance in Creative Singing Ability Score

Predictor	Partial correlation (pr)	*p*-Value
Step 1: *R* = 0.45, F(1,109) = 28.30, *p* < 0.001		
Spontaneous Speech Imitation Ability	0.45	< 0.001
Step 2: *R* = 0.52, F(1,108) = 8.65, *p* = 0.004		
Spontaneous Speech Imitation Ability	0.40	< 0.001
Fabel reading	0.27	0.004
Step 3: *R* = 0.54, F(1,107) = 4.03, *p* = 0.047		
Spontaneous Speech Imitation Ability	0.40	< 0.001
Fabel reading	0.27	0.018
Creative Task-Solving and Innovation Competence	0.19	0.047

Dependent variable: Creative Singing Ability Score

## Discussion

In this study, we addressed three questions: (1) Which musical, linguistic, and cognitive factors predict standard singing? (2) Do these predictors differ for creative singing? (3) What is the contribution of domain-general creative problem solving to creative vocal performance?

Based on the analyses, two main predictor profiles emerged: one for standard singing and another for creative singing. For each singing version, two stepwise multiple regression models were computed: (i) including linguistic and cognitive predictors only, and (ii) including both musical and non-musical predictors, to assess the incremental contribution of musical variables. In the models including musical predictors, both singing types were predicted by the composite musical ability score. However, standard singing was further predicted by training-related variables such as musical engagement and expressive reading ability, while creative singing, in contrast to standard singing, was predicted by spontaneous speech imitation, highlighting the role of linguistic–motor flexibility. When excluding musical predictors, the linguistic contributions become more pronounced: in the standard singing condition, expressive reading ability remained the sole significant predictor, emphasizing the role of language-based expressivity, whereas in the creative singing condition, a broader set of linguistic and cognitive skills—including speech imitation, expressive reading, and the concept Creative Task-Solving and Innovation Competence—emerged as significant predictors.

Focusing initially on standard singing, musical engagement, expressive reading, and the composite musical ability score emerged as significant predictors. Musical engagement reflected participants’ active involvement in musical activities, whereas expressive reading indexed audience-oriented vocal delivery characterized by expressive prosody, including intonation, rhythmic timing, emotional coloration, and intentional phrasing. That expressive reading emerged as a significant predictor indicates that audience-oriented expressive delivery in oral reading functions as a language-related pathway supporting vocal accuracy—consistent with work on reading prosody and fluent reading (Hudson et al., 2005; Kuhn & Stahl, 2003). In the present study, prosody is embedded within an audience-oriented expressive reading task.

When musical predictors were excluded, expressive fable reading was the only predictor that explained significant variance in singing ability. Raters evaluated expressive reading with audience orientation in mind, integrating intonation, rhythmic timing, emotional nuance, and intentional phrasing into a holistic score. Thus, while conceptually related to “reading prosody,” our measure operationalizes a broader construct of communicative expressivity. This finding supports the view that expressive reading—encompassing prosodic, emotional, and intentional aspects of spoken performance—bridges music and language through shared temporal and rhythmic processing (Stadler Elmer, 2021). It further aligns with evidence that musical and linguistic expertise mutually facilitate prosodic competence (Jansen et al., 2023). Overall, singing ability appears to emerge from reciprocal influences between musical engagement and expressive fluency, consistent with bidirectional transfer models linking language and music. However, another aspect must be considered: the second language task in this study, spontaneous speech imitation, did not contribute to explaining individual differences in general singing ability. Both expressive reading in the native language and singing a highly familiar melody may rely on familiarity-based routines rather than on-the-spot adaptation to novel material. This familiarity likely accounts for the stronger association between standard *Happy Birthday* singing and expressive reading, whereas imitating unfamiliar linguistic material requires greater flexibility and creativity to achieve high performance.

Creative singing revealed a distinct predictor profile: spontaneous speech imitation was the strongest cross-domain predictor, indicating that flexible auditory–motor mapping between language and music supports creative vocal performance. Speech imitation taps rapid auditory–motor mapping for unfamiliar phonetic–prosodic patterns—partly independent of expressive delivery—and can underwrite flexible melodic–prosodic recombination. When musical predictors were controlled, linguistic imitation and expressive reading—reflecting prosodic fluency and communicative expressivity—jointly accounted for significant variance in creative singing. This pattern suggests compensatory pathways: when musical expertise is controlled or limited, linguistic adaptability (imitation) and prosodic expressivity (expressive reading) account for additional variance, with domain-general creative problem solving making a modest, consistent contribution.

Within this framework, the constellation of predictors for creative singing can be interpreted through a sensorimotor lens. Spontaneous speech imitation reflects flexible auditory–motor coupling—the capacity to anticipate, reproduce, and adapt novel phonetic–prosodic patterns in real time—while expressive reading engages prosodic timing and pitch–rhythm modulation, indicating that continuous auditory feedback supports expressive fluency across both speech and song. These linguistic predictors capture perception–action coupling mechanisms that support flexible vocal control. The regression pattern thus provides convergent behavioral evidence that creative singing integrates auditory–motor precision (speech imitation), prosodic expressivity (expressive reading), and cognitive flexibility (creative problem solving). This interpretation aligns with models of auditory feedback regulation and predictive vocal control (Alemi et al., 2023), situating creative singing as a multimodal form of vocal behavior bridging music and language.

Paired models (with vs. without musical predictors) revealed a hierarchy: in the full model, composite musical ability absorbed shared variance; removing musical predictors unmasked independent contributions of speech imitation, expressive reading, and domain-general creativity—indicating distinct yet interacting levels of influence.

Musical ability predicts performance directly through auditory–motor precision and stylistic control, whereas domain-general creativity contributes to adaptive generativity—the capacity to recombine and elaborate vocal material in novel ways. Thus, creative flexibility becomes most visible at lower musical expertise, consistent with a compensatory mechanism whereby domain-general and linguistic resources sustain creative output.

This interpretation aligns with network-neuroscience models proposing that creativity involves dynamic reconfiguration of domain-specific and domain-general networks according to task demands (Beaty et al., 2016). Reading such compensatory effects explain why some individuals achieve high creative-singing performance despite modest musical aptitude—they may draw on linguistic or expressive resources or a general creative mindset that substitutes for formal musical structure (Baer, 2016; Christiner & Reiterer, 2013; Christiner & Reiterer, 2015). From this perspective, creative singing thus constitutes a dynamic system in which varying configurations of musical, linguistic, and cognitive resources can yield comparable creative outcomes. This dynamic perspective aligns with neurocognitive models of improvisation, in which adaptive auditory–motor feedback loops interact with higher-order control networks to support flexible vocal creativity (Liu et al., 2012; Loui, 2018; Schiavio & Benedek, 2020). Such coupling between monitoring and generative systems provides a mechanistic substrate for the dual-pathway dynamics observed in our data.

Our findings point to two complementary yet partly compensatory pathways of vocal performance:

1. standard singing, grounded in musical engagement, expressive reading, and composite musical competence, and.
2. creative singing, supported by linguistic imitation, expressive reading, and domain-general creative flexibility.

These profiles suggest multiple routes to vocal creativity, indicating that prosodic expressivity and linguistic adaptability may complement or partly substitute for musical expertise, with potential implications for applied and pedagogical contexts (Christiner & Reiterer, 2013; Christiner & Reiterer, 2015; Loui, 2018; Schiavio & Benedek, 2020).

Educationally, expressive reading and musical performance appear mutually reinforcing. Integrative tasks—for example, improvisational call-and-response exercises that alternate speech rhythms and sung motifs—can train timing, phrasing, and communicative intent concurrently (Stadler Elmer, 2021). Expressive reading activities that cultivate expressive prosody—including intonational nuance, rhythmic flexibility, and emotional modulation—may strengthen vocal timing and affective expression in singing. (Hudson et al., 2005; Kuhn & Stahl, 2003). Conversely, singing-based exercises can enhance oral fluency, prosodic sensitivity, and phonological awareness in reading (Gordon et al., 2015; Patel, 2011). Curricula that intentionally link expressive and musical components could therefore foster linguistic and musical development more effectively than isolated forms of practice.

Creative singing further extends these opportunities by emphasizing improvisation, variation, and spontaneous imitation (Berkowitz, 2010; Loui, 2018). Pedagogically, it may offer an accessible entry point for learners with limited formal training, encouraging engagement through vocal experimentation and verbal creativity.

From an applied perspective, the observed links between speech imitation, expressive reading, and creative singing suggest possible relevance for contexts in which vocal flexibility and prosodic control are central—such as language learning, communication training, or voice pedagogy (Alemi et al. 2023; Christiner et al. 2021, 2022a, b, c; Prather 2013).

Integrating music, language, and creativity holds both theoretical and applied significance. Our findings support the view that these domains are interconnected through shared auditory–motor mechanisms (Hutchins & Moreno, 2013; Zatorre et al., 2007) yet may flexibly compensate for one another depending on individual resource profiles. This integrative perspective helps explain how expressive vocal performance can emerge even when formal musical expertise is limited (Beaty et al., 2016; Schiavio et al., 2023). Future research may extend these findings by examining longitudinal developments and incorporating experimental or neurophysiological approaches to further explore the mechanisms linking musical, linguistic, and creative factors in vocal performance.

## Materials and methods

### Participants

A sample of 111 participants were recruited for this study, all of whom provided informed consent and participated voluntarily. The cohort comprised 74 female, 37 male, and one non-binary individual. Regarding musical training, 39 participants reported no prior instrumental acquisition, whereas 72 indicated having learned at least one musical instrument during their lifetime. To assess participants’ current training status, we used self-reported practice hours of singing and instrument playing over the past three years and number of instruments played, from which we computed a composite Musical Engagement Score (see Materials and method section). Additionally, 12 participants reported having received professional vocal training during their lives. The native languages of the participants was German and all participants received at least eight years of English instruction as a foreign language in school. The mean age was 32,8 years (SD = 13,75). Recruitment occurred via online platforms and university channels, with inclusion criteria specifying a minimum age of 18 years.

### Singing ability

Singing ability was assessed using two different tasks, both performed in English: First, participants were asked to sing the song “Happy Birthday” as beautifully as possible. Second, they were instructed to sing “Happy Birthday” again, this time as creatively as possible, to evaluate their creative singing skills. For this, participants were additionally informed that they could freely change the rhythmic or melodic qualities of “Happy Birthday,” but should still keep in mind that the song ought to sound pleasant to listeners. We used the “Happy Birthday” singing task because it is well-suited to assess individual differences in singing ability for both professional and non-professional singers (Dalla Bella & Berkowska, 2009; Dalla Bella et al., 2007), and it has been employed in multiple studies where it proved highly effective for this purpose (Christiner et al. 2022a, b, c; Christiner & Reiterer, 2013; Christiner et al. 2022a, b, c).

All vocal recordings were edited using standard audio-editing software. Leading and trailing silence was removed and overall amplitude was normalized to ensure comparable playback levels across stimuli. No pitch correction, time-stretching, or other manipulations affecting pitch or timing were applied. The resulting audio excerpts were anonymized and presented to the raters in randomized order.The singing performances of the participants were carefully reviewed and edited, with loudness levels standardized prior to uploading to the rating platform. Subsequently, professional musicians (*n* = 8) evaluated both singing tasks. The raters were provided with the rating criteria, along with the detailed instructions previously given to the participants. The first “Happy Birthday” singing task (referred to as Singing Ability Score) was assessed across four dimensions—melodic accuracy, rhythmic accuracy, vocal range, and voice quality—based on established criteria from prior research (Christiner et al. 2022a, b, c). These dimensions were combined into a composite score. The second creative singing task (referred to as Creative Singing Ability Score) was rated according to the perceived creativity of the “Happy Birthday” performances by the listeners and represents a single score.

Inter-rater reliability was determined for all rating criteria separately using intra-class correlation coefficients (ICCs), which demonstrated a high level of agreement well above the commonly accepted threshold of 0.7 (see Supplementary Tables S1-S5).

### Aggregation of objective musical performance and self-reported ability

To capture a comprehensive measure of the musical skills of the participants, we computed a composite score combining three objective performance musicality measures (parts of the LongGold Test Battery https://longgold.org/) and three self-estimated musical ability concepts. Given the strong conceptual relation and the expected statistical correlation between these measures, the resulting composite represents an integrated index of musical competence, encompassing both demonstrated performance and subjective self-evaluation. The three performance subtests of the LongGold Test Battery were first z-transformed and aggregated into a single performance score. Likewise, the multi-item self-estimation scale was z-transformed to form a self-assessment score (see also Sect. 3.2.4). Finally, these two scores—performance and self-assessment—were combined following principal components analysis (PCA) confirming a single-factor structure (eigenvalue = 3.46, 58% variance explained; loadings ≥ 0.65; KMO = 0.80; see Supplemental Material S8 for details, including intercorrelations, loadings, communalities, and Cronbach’s α = 0.83) by averaging their z-values to create an overall composite measure of musical ability (referred to as Composite Musical Ability Score). The individual subtests and the self-estimation scale are described in detail below.

### Computerised Adaptive Beat Alignment Test (CA-BAT)

Individual differences in beat perception as a component of musical ability, was measured by the Computerised Adaptive Beat Alignment Test (CA-BAT), a psychometric instrument developed by (Harrison & Müllensiefen, 2018). Analogous to the approach used for deriving the Composite Musical Ability Score, the CA-BAT provides an objective, performance-based measure that integrates key methodological innovations in psychometric testing. The CA-BAT builds upon the classical Beat Alignment Test (BAT) and adopts a two-alternative forced-choice (2-AFC) paradigm: Participants listen to two versions of a five-second musical excerpt, each overlaid with a beep track. One version is metrically aligned with the underlying musical beat (target), whereas the other is systematically misaligned (lure). The task is to identify the better-aligned version. The CA-BAT incorporates three major psychometric advancements: (1) Item Response Theory (IRT) allows ability estimates to be comparable across different item sets; (2) Computerised Adaptive Testing (CAT) dynamically selects the most informative items based on the participant’s current ability estimate; and (3) Automatic Item Generation enables the construction of extensive item banks with empirically validated control over item difficulty, primarily modulated by the degree and direction of beat-track displacement. Test items are drawn from a bank of 32 musical excerpts across various genres, with canonical beat locations determined by expert drummer performance. The adaptive algorithm tailors item selection in real time, thereby increasing measurement precision while minimising test duration.

Validation studies have shown that the CA-BAT exhibits strong construct validity, with moderate correlations to musical training (*r* ≈ 0.40) and good test–retest reliability in controlled settings (*r* = 0.67). However, reliability is substantially reduced in uncontrolled online environments, highlighting the importance of standardized test administration. As such, the CA-BAT constitutes a robust tool for examining individual variability in beat perception and its neural and behavioral correlates. Test materials and implementation guidelines are openly available via Zenodo (https://zenodo.org/records/1299599).

### Melodic Discrimination Test (MDT)

To assess melodic discrimination ability as a core component of musical perception, we employed the Melodic Discrimination Test (MDT), developed by (Harrison et al., 2017). The MDT is a computerised adaptive assessment tool grounded in contemporary psychometric methodologies, including Item Response Theory (IRT), Computerised Adaptive Testing (CAT), and Automatic Item Generation (AIG). It offers a precise and time-efficient measure of an individual’s capacity to detect local melodic deviations.

Each trial follows a three-alternative forced-choice (3-AFC) paradigm, in which participants are presented with three short melodic sequences. Two of these (foils) are identical aside from a global transposition of one semitone up or down, while the third sequence (target) contains a single pitch alteration that disrupts the underlying melodic contour. Participants are instructed to identify the deviant sequence. Stimuli are generated algorithmically from a statistical model trained on a corpus of Irish folk melodies, ensuring ecological validity. Test items consist of 3 to 16 notes and systematically vary in difficulty, which is determined by musical-structural parameters such as the presence of contour violations, tonal regularity, and melodic expectancy. Transpositions are implemented to eliminate reliance on absolute pitch cues, thereby isolating relative pitch processing abilities.

The MDT is administered via the psychTestR online platform and adapts item selection in real time based on participants’ performance, thereby optimising reliability and test economy. Empirical validations have demonstrated strong psychometric properties, including high internal consistency (Cronbach’s α > 0.80) and substantial construct validity, with robust associations to formal musical training and related auditory-cognitive skills. As such, the MDT constitutes a valuable instrument for examining individual differences in melodic perception and their cognitive underpinnings.

### Mistuning Perception Test (MPT)

To assess listeners’ sensitivity to vocal mistuning, we employed the Mistuning Perception Test (MPT), an adaptive and ecologically valid measure introduced by (Larrouy-Maestri et al., 2019).

Sensitivity to vocal pitch inaccuracies was assessed using the Mistuning Perception Test (MPT), an adaptive listening task designed to measure the ability to detect pitch deviations in sung music. The test is based on short audio excerpts (6–12 s) taken from commercially released pop recordings. For each excerpt, the vocal track is either presented in its original form or artificially pitch-shifted relative to the instrumental accompaniment.

Each trial follows a two-alternative forced-choice (2AFC) procedure. Participants listen to two versions of the same musical excerpt presented in random order—one original and one containing a manipulated vocal pitch—and are asked to identify the version in which the singing is mistuned.

During the development phase, the test was administered in a fixed-format version to a large sample of listeners (*N* = 333) in order to calibrate item difficulty using explanatory item response modeling. Based on these data, an adaptive version of the test was subsequently implemented. In this adaptive format, the difficulty of the presented items is dynamically adjusted depending on the participant’s responses, allowing efficient and precise estimation of mistuning perception ability.

Validation with an additional sample of participants representing a broad range of musical backgrounds demonstrated good test–retest reliability as well as satisfactory convergent and divergent validity. The MPT therefore provides an efficient and ecologically valid tool for assessing individual differences in sensitivity to vocal pitch deviations.

### Self-assessment measures of singing, melodic memory, and musical skills participants

To assess individual differences in musical ability, we developed three multi-item self-assessment scales measuring participants’ perceived abilities in singing, performing musical tasks, and memorizing melodies.

The singing self-estimation scale, consisting of 7 items, asked participants to rate their perceived competence specifically related to the singing task, capturing both task-specific and more general aspects of singing skills. The musical ability self-estimation scale, consisting of 6 items, measured participants’ subjective evaluation of their overall performance in general musical tasks, reflecting perceived musical skills beyond the singing context. The melody memory scale is composed of 6 items and assessed individual differences in the tendency to attend to, remember, and mentally replay melodies during music listening.

Each scale comprised multiple items rated on an 11-point Likert scale ranging from 0 (strongly disagree/not at all) to 10 (strongly agree/completely), with higher scores indicating stronger presence of the respective construct. The individual items and details of reliability analyses demonstrating good internal consistency (Cronbach’s α > 0.7) are provided in Supplement Sect. 2. Additional methodological details are provided in Supplementary Material S8.

Before aggregation, the scores of the three self-assessment scales were z-transformed to ensure comparability and then combined to form an overall composite measure representing subjective musical competence.

Participants completed the Composite Musical Ability Measurements, which encompassed a range of tasks designed to objectively assess distinct facets of musical ability, including a singing task. Immediately following each task, participants were asked to rate their own performance and abilities using corresponding multi-item self-assessment scales. This procedure was implemented to elicit more accurate and context-sensitive self-evaluations by placing the subjective assessment in direct temporal proximity to the objective task. In doing so, the design aimed to enhance the ecological validity of the self-report data by anchoring participants’ judgments in their immediate task experience.

### Musical engagement

We also collected information on participants’ active musical engagement. Participants reported the number of musical instruments ever played (M = 1.36, SD = 1.31), hours per week (hpw) of instrument practice (M = 1.62, SD = 2.99), and regular singing (M = 2.25, SD = 3.47; *N* = 111 each), considering the past three years retrospectively. As instrument count and practice hours were highly correlated, these were summed and – together with singing hpw – z-transformed (all M = 0, SD = 1) before averaging to form the composite Musical Engagement Score.

### Musical socialisation and relevance

To capture the socialization process related to music, we developed a comprehensive approach combining two complementary measures. First, we constructed a new multi-item scale assessing the subjective relevance of music in participants’ lives, reflecting their personal valuation and importance of music. Early engagement with singing and music in childhood is considered a key foundation for later musical development. Regular singing during formative years reflects informal training and active involvement in music, especially for individuals without access to formal music education. The childhood singing behavior scale thus captures an essential aspect of early musical experience.

Second, we incorporated a previously developed questionnaire measuring singing behavior during childhood (Christiner et al. 2022a, b, c), which serves as an objective marker of early musical socialization. Participants rated each item on an 11-point Likert scale ranging from 0 (strongly disagree/not at all) to 10 (strongly agree/completely).

To evaluate the reliability of the multi-item scale assessing music relevance, a reliability analysis was conducted, demonstrating that the internal consistency exceeds the commonly accepted threshold of 0.7 (Cronbach’s α > 0.7). The items and questions are presented in the supplementary materials (see Sect. 3).

Before aggregation, both components—the subjective importance of music and early childhood singing behavior—were standardized using z-transformation to ensure comparability. These standardized scores were then combined into a single composite measure, the ‘Musical Socialisation and Relevance Score,’ capturing the multifaceted nature of musical socialization across the lifespan.

### Passively listening to music

Passive listening to music represents a widespread and general form of musical engagement in everyday life. Although it is a less active behavior compared to playing an instrument or singing, it is important to assess because of its potential influence on cognitive processes and overall musical involvement. Therefore, we developed and included a dedicated multi-item scale to capture this dimension of music listening behavior. Passive music listening was assessed using a four-item scale. The composite measure derived from these items is henceforth referred to as the PassiveMusicListen score.

Participants rated each item on an 11-point Likert scale ranging from 0 (strongly disagree/not at all) to 10 (strongly agree/completely). To evaluate the reliability of the multi-item scale assessing music relevance, a reliability analysis was conducted, demonstrating that the internal consistency exceeds the commonly accepted threshold of 0.7. The items and reliability analysis are presented in the supplementary materials (see Sect. 4).

### Multi-item scale concept creative task-solving and innovation competence

As we assessed creative singing ability, we also wanted to include a questionnaire specifically addressing whether participants perceive themselves as creative and innovative when tackling unfamiliar tasks.

To capture individual creativity in dealing with new challenges, we used a self-report questionnaire developed specifically for this study. The multi-item scale consists of 10 items targeting individual tendencies toward creative cognitive behavior when completing novel or unusual tasks. The statements refer to general tendencies in everyday situations that require finding solutions, such as a preference for generating novel ideas, acting spontaneously, or approaching challenges independently.

Participants rated each item on an 11-point Likert scale ranging from 0 (strongly disagree/not at all) to 10 (strongly agree/completely). Reliability was assessed using Cronbach’s alpha, which exceeded the commonly accepted threshold of 0.7. The exact values and individual items are provided in the supplemental section (see supplement Sect. 5).

### Language measures

To capture the linguistic dimensions relevant to vocal creativity, we included two complementary language measures targeting expressive prosody and speech imitation. Both tasks were designed to assess skills that parallel the musical and auditory–motor mechanisms underlying singing. The first task, an expressive reading of the fable *The North Wind and the Sun*, measured prosodic fluency and expressive prosody in natural speech. The second task assessed participants’ ability to spontaneously imitate unfamiliar languages (Tagalog and Mandarin), thereby capturing flexibility in auditory–motor mapping and phonological adaptation. Together, these measures provide a comprehensive index of linguistic performance, encompassing both the prosodic and imitative aspects of speech that are theoretically linked to vocal and creative singing ability.

### Reading the fable north wind and the sun

To assess individual differences in reading ability, we employed a reading task in which participants were asked to read the German version of the well-known fable The North Wind and the Sun. They were instructed to read the fable to the best of their ability, specifically with the intention of captivating and holding the attention of children as their listeners. This task was designed to capture not only their reading fluency but also their expressive and engaging oral presentation skills. To evaluate the performances of the reading task, we employed established methods and recruited raters who had to fulfil two main criteria. They had to be German native speakers as well as come from a linguistic background.

For the ratings, the participants’ recordings were uploaded to an online platform. The raters were instructed to evaluate the participants’ performances and were provided with the same information and instructions as the participants. Then, the raters were asked to assess how well the participants read the fable in order to capture and hold the attention of children as listeners. The raters used a scale ranging from 0 (“min”) to 10 (“max”). To assess the reliability of the ratings, intraclass correlation coefficients (ICCs) were computed. The results showed that the reliability of the ratings was above the threshold of 0.7 (see Supplementary Table S6).

#### Language ability measure

To assess individual differences in the ability to imitate unfamiliar languages, we employed language stimuli previously used to measure language aptitude (Christiner et al. 2021, 2022a, b, c, 2023). These tasks are designed to evaluate how quickly and accurately individuals can adapt to novel linguistic material, thereby requiring both intuitive and flexible language processing skills.

For the language performance tasks, we used stimuli consisting of nine and eleven syllables, spoken by three male and three female native speakers of two different languages Tagalog and Mandarin —languages rarely spoken by German native speakers, which minimizes the likelihood of prior language contact influencing performance.

Participants listened to each language sample three times, with each presentation separated by a 50 ms pause, before being asked to repeat the phrases as accurately as possible.

Prior to the main task, participants completed a familiarization phase to ensure they understood the procedure. During this phase, they listened to Tagalog samples distinct from those used in the main experiment, allowing them to become acquainted with the task format. Following familiarization, participants were asked to imitate six Tagalog and Mandarin samples, with the order of presentation randomized for each individual. The participants’ recordings were subsequently prepared for further analysis: all recordings were checked, and normalized for loudness. For the evaluation, native speakers assessed the participants’ performances.

To evaluate the performances, we employed established methods and recruited native speakers of Tagalog to assess individual differences in language performances (Christiner et al. 2021, 2022a, b, c). The participants’ recordings were uploaded to a dedicated online platform specifically designed for this purpose. A group of raters, all native speakers of the respective target languages (Mandarin, *n* = 40; Tagalog, *n* = 16), was recruited to evaluate these recordings. Each rater assessed the overall performance of the language stimuli using a continuous rating scale ranging from 0 (“minimum”) to 10 (“maximum”) in the corresponding language. To evaluate the consistency and reliability of the ratings across raters, intraclass correlation coefficients (ICCs) were computed. The results showed that the reliability of the ratings was above the threshold of 0.7 (see Supplementary Tables S7-S18).

Furthermore, given previous research indicating that language typology has little influence on imitation tasks when individuals are learning novel material, a composite score (Spontaneous Speech Imitation Ability) combining the ratings from both Mandarin and Tagalog was created to more robustly assess overall imitation ability across languages. This composite score reflects participants’ performance in the initial imitation tasks, capturing their ability to reproduce unfamiliar speech immediately upon first exposure.

### Statistical analysis

The statistical analyses were conducted in a series of consecutive steps, each addressing a distinct research aim. In the first stage, we computed Pearson correlation coefficients to examine the bivariate relationships between all core variables. This included the relationships between Singing Ability and Creative Singing Ability and a broad set of musical, creative, and language-related measures. Musical measures comprised objective perception tasks (Computerised Adaptive Beat Alignment Test [CA-BAT], Melodic Discrimination Test [MDT], Mistuning Perception Test [MPT]) and self-reported abilities (Singing Self-Estimation, Musical Ability Self-Estimation, Melody Memory). In addition, indices of musical engagement (weekly hours of singing, weekly hours of instrumental practice, number of instruments played), musical socialisation and relevance, and passive music listening were considered. Creative and cognitive measures included the Creative Task-Solving and Innovation scale, while language-related variables comprised expressive fable reading scores and spontaneous speech imitation scores (Mandarin and Tagalog, combined into a composite).

Based on the inspection of the correlation matrix, the second stage of the analysis involved two separate stepwise multiple regression models. Because musical and linguistic predictors were substantially intercorrelated, we report regression models both with and without musical predictors. This approach follows established methodological recommendations (Budescu, 1993; Cohen, 2013; Dormann et al., 2013; Johnson, 2000). Dominant predictors such as musical ability can absorb large proportions of shared variance, which may overshadow the contribution of other relevant variables. By estimating additional models excluding musical predictors, the incremental role of linguistic and cognitive factors can be assessed more accurately. Models excluding musical predictors thus allow for a clearer assessment of the incremental contribution of linguistic and cognitive factors. In the first model, Singing Ability was entered as the dependent variable, while all remaining measures were treated as potential predictors. In the second model, Creative Singing Ability served as the dependent variable with the same set of predictors considered. This procedure allowed for the identification of distinct sets of variables that best predict Singing Ability and Creative Singing Ability. As the correlation matrix indicated that musical variables were the strongest predictors of singing ability, regression models were calculated both including and excluding these variables. Presenting both approaches is methodologically informative: when musical predictors are included, they tend to dominate the models and may overshadow other predictors. When musical predictors are excluded, the relative contribution of linguistic variables can be more accurately assessed, highlighting their role in the absence of musical influence.

## Supplementary Information

Below is the link to the electronic supplementary material.


Supplementary Material 1 (PDF 623 KB)


## Data Availability

The datasets generated and analysed during the current study are available from the corresponding author upon reasonable request due to privacy and ethical restrictions.
