# Supplementary material for: Sounds of creativity: musical, creative, and language factors associated with singing and creative singing
Source: Psychol Res. 2026 Jul 6;90(4):128. doi: 10.1007/s00426-026-02346-x (PMC13337679; doi:10.1007/s00426-026-02346-x)
Supplement: Supplementary file 1 — Supplementary Material 1 (PDF 623 KB) [file 426_2026_2346_MOESM1_ESM.pdf]

Supplementary Information for: Sounds of Creativity: Musical, Creative, and Language Factors Associated with Singing and Creative Singing"

This Supplementary Information accompanies the article and provides extended descriptions of the experimental materials, procedures, and analyses reported in the main text. The document includes detailed task descriptions, additional statistical models, and Supplementary Tables S1–S20, summarizing interrater reliability (intraclass correlation coefficients), internal consistencies of the multi-item scales, and extended regression outputs for all musical, linguistic, and creative variables. All materials and data relevant to the reported findings are presented here to ensure methodological transparency and reproducibility.

1. Intraclass correlation coefficients singing tasks

Tables S1 to S4 display the intraclass correlation coefficients (ICCs) of the rating criteria obtained for the first "Happy Birthday" singing. Table S5 shows the intraclass correlation coefficients (ICCs) obtained for the second Happy Birthday singing. These coefficients quantify the degree of agreement among raters, reflecting the reliability and consistency of the ratings assigned to participants' performances. Following regression analyses, participants with large, standardized residuals exceeding ±3 were identified and excluded to enhance the robustness of the final analysis. Consequently, while the ICC values remained unchanged, the sample size—and thus the degrees of freedom—differed slightly between the initial ICC calculation and the final analysis reported in the Results section.

Table S1. Intraclass correlation coefficients: Singing Happy Birthday 1 (melody)

		95% Confidence Interval		F Test with True Value 0			
	Intraclass Correlation	Lower Bound	Upper Bound	Value	df1	df2	Sig
Average Measures	.86	.77	.91	12.91	122	976	0,001

Table S2 . Intraclass correlation coefficients: Singing Happy Birthday 1 (quality of voice)

		95% Confidence Interval		F Test with True Value 0			
	Intraclass Correlation	Lower Bound	Upper Bound	Value	df1	df2	Sig
Average Measures	.82	.69	.89	11.03	122	976	0,001

Table S3. Intraclass correlation coefficients: Singing Happy Birthday 1 (rhythm)

	Intraclass Correlation	95% Confidence Interval		F Test with True Value 0			
		Lower Bound	Upper Bound	Value	df1	df2	Sig
Average Measures	.70	.49	.81	7.20	122	976	0,001

Table S4. Intraclass correlation coefficients: Singing Happy Birthday 1 (vocal range)

	Intraclass Correlation	95% Confidence Interval		F Test with True Value 0			
		Lower Bound	Upper Bound	Value	df1	df2	Sig
Average Measures	.88	.81	.93	15.24	122	976	0,001

Table S5. Intraclass correlation coefficients: Singing Happy Birthday 2 (creativity)

	Intraclass Correlation	95% Confidence Interval		F Test with True Value 0			
		Lower Bound	Upper Bound	Value	df1	df2	Sig
Average Measures	.77	.64	.84	6.58	122	854	0,001

2. Self-Assessment Measures of Singing, Melodic Memory, and Musical Skills Participants

2.1 Multi-item scales concept singing self-estimation scale

Below the items of the multi-item scale concept singing self-estimation are presented. The items were each rated on an 11-point Likert scale ranging from 0 (strongly disagree/not at all) to 10 (strongly

agree/completely). The internal consistency of the scale was assessed using Cronbach's alpha (6 items), yielding a value of α = 0.91, indicating acceptable reliability.

a. I sing the notes of melodies correctly right away.
b. I have a very good sense for singing.
c. Others say that I am a good singer.
d. I sang the song I sang before very beautifully.
e. When I sing, I often change the melody on familiar songs.
f. I can easily change my voice pitch to sing songs higher or lower.
g. I create songs in my mind.

2.2 Multi-item scales concept musical ability self-estimation scale

Below the items of the multi-item scale concept musical ability self-estimation are presented. The items were each rated on an 11-point Likert scale ranging from 0 (strongly disagree/not at all) to 10 (strongly agree/completely). The internal consistency of the scale was assessed using Cronbach's alpha (6 items), yielding a value of α = 0.93, indicating acceptable reliability.

a. Musicality tasks like I did before are not difficult to solve.
b. Rhythm changes are difficult to hear. But I think I can do it very well.
c. I hear when music or music parts sound wrong.
d. I can tell if someone is hitting the notes.
e. I think I did very well on the music tasks I did before.
f. I have a very good sense of rhythm.

2.3 Multi-item scales concept melody memory scale

Below the items of the multi-item scale concept melody memory are presented. The items were each rated on an 11-point Likert scale ranging from 0 (strongly disagree/not at all) to 10 (strongly agree/completely). The internal consistency of the scale was assessed using Cronbach's alpha (6 items), yielding a value of α = 0.78, indicating acceptable reliability.

a. Remembering melodies is very easy for me.
b. When I hear a song on the radio, even hours later I can still hear the melody imaginary.
c. I don't remember the melody of songs I don't like.
d. The first thing I remember about a song is the melody.
e. I listen to music attentively every day.
f. Distinguishing melodies is very easy for me.

3. Music socialisation and relevance

This section outlines the single items of both multi-item scales concepts singing behaviour during childhood and musical relevance**.** The items were each rated on an 11-point Likert scale ranging from 0 (strongly disagree/not at all) to 10 (strongly agree/completely). For assessing the internal consistency a reliability analysis was conducted. The internal consistency of singing behaviour during childhood was assessed using Cronbach's alpha (10 items), yielding a value of α = 0.92, indicating acceptable reliability. The internal consistency of musical relevance was assessed using Cronbach's alpha (10 items), yielding a value of α = 0.78, also indicating acceptable reliability.

3.1 Singing behaviour during childhood

a. As a child I enthusiastically joined in with the singing at church and similar events whenever the possibility arose.
b. As a child I was encouraged to sing by my caretakers and we sang together on a weekly basis even if there were no special events.
c. As a child I enjoyed singing in a choir, with friends, at Christmas, birthdays, or at similar occasions.
d. As a child I sang very often since I wanted to become a musician or singer.
e. As a child I used to sing whenever I could such as in the bathroom, in the car, when I played with friends
f. As a child I liked being a member of our school choir, or would have liked being a member or a school choir.
g. As a child I enjoyed singing a song that had been played to me (e.g., in the radio).
h. As a child I used to sing more often than my friends.
i. In my childhood singing played a major role
j. As a child my parents did not sing a lot with me.

3.2 Musical relevance

a. I would not describe myself as a musician.
b. I grew up with a lot of music
c. When I was a child, my parents listened to music all the time.
d. Music has always been an important part of my life.
e. I listened to someone play an instrument at least once a week as a child.

4. Passively Listening to Music

The multi-item scale assessing passive music listening comprised four items, each rated on an 11-point Likert scale ranging from 0 (strongly disagree/not at all) to 10 (strongly agree/completely). The internal consistency of musical relevance was assessed using Cronbach's alpha, yielding a value of $\alpha = 0.83$, also indicating acceptable reliability.
Examples of items include:

a. When I listen to music while I study, it's much easier for me to keep going.
b. I believe that I learn things more easily when I listen to music.
c. I hear music in the background all the time.
d. I listen to more than 5 hours of music a day (e.g. radio, Spotify, or similar).

5. Multi-item Scale Concept Creative Task-Solving and Innovation Competence

Below the items of the multi-item creative task-solving and innovation competence are presented. The items were each rated on an 11-point Likert scale ranging from 0 (strongly disagree/not at all) to 10 (strongly agree/completely). The internal consistency of the scale was assessed using Cronbach's alpha (10 items), yielding a value of $\alpha = 0.86$, indicating acceptable reliability.

a. When I have difficulties solving tasks I come up with something alternative.
b. I prefer to look for my own solutions instead of following the given path.
c. I often think about how to solve tasks without following the advice of others.
d. I find that I am very creative in finding solutions.

e. Often no one notices that I manage to get things done even though I didn't know how it worked.
f. I'm very innovative when it comes to getting things done.
g. I can come up with something spontaneously whenever I need it.
h. Others say that I think of unusual things to approach something.
i. I am very flexible.
j. I think that I can solve tasks well without thinking for a long time.

6. Intraclass correlation coefficients: Reading the Fable Nord Wind and Sun

Table S6. Intraclass correlation coefficients: Reading the Fable Nord Wind and Sun

Table S18 displays the intraclass correlation coefficients (ICCs) of the Fable reading Nord Wind and the sun. Intraclass correlation coefficients (ICCs) were initially calculated using the full sample to assess the reliability of participant performance ratings. Following regression analyses, participants with large standardized residuals exceeding ±3 were identified and excluded to enhance the robustness of the final analysis. Consequently, while the ICC values remained unchanged, the sample size—and thus the degrees of freedom—differed slightly between the initial ICC calculation and the final analysis reported in the Results section.

Table S6. Intraclass correlation coefficients: Fable reading Nord Wind and the sun

		95% Confidence Interval		F Test with True Value 0			
	Intraclass Correlation	Lower Bound	Upper Bound	Value	df1	df2	Sig
Average Measures	.70	.56	.80	4.38	117	468	0,001

7. Intraclass correlation coefficients of the language ability measures

Tables S7 to S18 display the intraclass correlation coefficients (ICCs) of the Tagalog and Mandarin ratings. Intraclass correlation coefficients (ICCs) were initially calculated using the full sample to assess the reliability of participant performance ratings. Following regression analyses, participants with large standardized residuals exceeding ±3 were identified and excluded to enhance the robustness of the final analysis. Consequently, while the ICC values remained unchanged, the sample size—and thus the degrees of freedom—differed slightly between the initial ICC calculation and the final analysis reported in the Results section.

Table S7. Intraclass correlation coefficients: Tagalog sentence (A1)

		95% Confidence Interval		F Test with True Value 0			
	Intraclass Correlation	Lower Bound	Upper Bound	Value	df1	df2	Sig
Average Measures	.92	.89	.94	16.71	117	1755	0,001

Table S8. Intraclass correlation coefficients: Tagalog sentence (A2)

		95% Confidence Interval		F Test with True Value 0			
	Intraclass Correlation	Lower Bound	Upper Bound	Value	df1	df2	Sig
Average Measures	.94	.92	.96	20.39	118	1770	0,001

Table S9. Intraclass correlation coefficients: Tagalog sentence (B1)

		95% Confidence Interval		F Test with True Value 0			
	Intraclass Correlation	Lower Bound	Upper Bound	Value	df1	df2	Sig
Average Measures	.92	.89	.94	20.39	116	1856	0,001

Table S10. Intraclass correlation coefficients: Tagalog sentence (B2)

	Intraclass Correlation	95% Confidence Interval		F Test with True Value 0			
		Lower Bound	Upper Bound	Value	df1	df2	Sig
Average Measures	.94	.93	.96	21.99	119	1785	0,001

Table S11. Intraclass correlation coefficients: Tagalog sentence (C1)

	Intraclass Correlation	95% Confidence Interval		F Test with True Value 0			
		Lower Bound	Upper Bound	Value	df1	df2	Sig
Average Measures	.92	.89	.94	18.28	121	1815	0,001

Table S12. Intraclass correlation coefficients: Tagalog sentence (C2)

	Intraclass Correlation	95% Confidence Interval		F Test with True Value 0			
		Lower Bound	Upper Bound	Value	df1	df2	Sig
Average Measures	.92	.90	.95	18.33	121	1815	0,001

Table S13. Intraclass correlation coefficients: Mandarin sentence (A1)

	Intraclass Correlation	95% Confidence Interval		F Test with True Value 0			
		Lower Bound	Upper Bound	Value	df1	df2	Sig
Average Measures	.97	.96	.98	43.44	119	4641	0,001

Table S14. Intraclass correlation coefficients: Mandarin sentence (A2)

	Intraclass Correlation	95% Confidence Interval		F Test with True Value 0			
		Lower Bound	Upper Bound	Value	df1	df2	Sig
Average Measures	.97	.97	.98	55.53	118	4602	0,001

Table S15. Intraclass correlation coefficients: Mandarin sentence (B1)

	Intraclass Correlation	95% Confidence Interval		F Test with True Value 0			
		Lower Bound	Upper Bound	Value	df1	df2	Sig
Average Measures	.97	.96	.98	53.05	120	4680	0,001

Table S16. Intraclass correlation coefficients: Mandarin sentence (B2)

	Intraclass Correlation	95% Confidence Interval		F Test with True Value 0			
		Lower Bound	Upper Bound	Value	df1	df2	Sig
Average Measures	.97	.97	.98	51.156	117	4563	0,001

Table S17. Intraclass correlation coefficients: Mandarin sentence (C1)

	Intraclass Correlation	95% Confidence Interval		F Test with True Value 0			
		Lower Bound	Upper Bound	Value	df1	df2	Sig
Average Measures	.97	.96	.98	57.48	119	4641	0,001

Table S18. Intraclass correlation coefficients: Mandarin sentence (C2)

		95% Confidence Interval		F Test with True Value 0			
	Intraclass Correlation	Lower Bound	Upper Bound	Value	df1	df2	Sig
Average Measures	.97	.96	.98	45.57	119	4641	0,001

8. **Justification of the Musical Ability Composite Score**
We conducted a principal components analysis (PCA) to examine whether the objective musical performance measures and self-reported musical ability formed a unidimensional construct, followed by an assessment of internal consistency using Cronbach's alpha.

Suitability Tests
The Kaiser-Meyer-Olkin measure of sampling adequacy was .80 (good), and Bartlett's test of sphericity was significant, $\chi^2(15) = 276$, $p < .001$, confirming the data's suitability for PCA (N = 111 listwise).

Intercorrelations
Pearson correlations among the six measures ranged from .34 to .81 (all $p < .001$, 1-tailed; determinant = .07), indicating moderate to strong interrelations appropriate for aggregation. See Table S19 (Supplemental Material) for the full matrix.

Table S19. Pearson correlations

Measure	BAT	MDT	MPT	Self-Musical	Self-Singing	Self-Melodic
BAT ability	1.00					
MDT ability	.51**	1.00				
MPT ability	.43**	.55**	1.00			
Self-Musical Skills	.40**	.37**	.42**	1.00		
Self-Singing	.34**	.50**	.47**	.68**	1.00	
Self-Melodic Memory	.44**	.51**	.37**	.71**	.64**	1.00

Note. Pearson correlations; **p < .001 (1-tailed).

Internal Consistency
Cronbach's α for the six raw-score items was .83 (Item-total correlations .50–.74; α if item deleted .76–.82), indicating good reliability for the composite (see Table S20 below).

Table S20. Item analysis and internal consistency (Cronbach's α) for the Composite Musical Ability Score.

Item Deleted	Item-Total r	α if Deleted
BAT	0.50	0.82
MDT	0.59	0.81
MPT	0.54	0.82
Self_Music	0.74	0.76
Self_Singing	0.73	0.78
Melody Memory	0.74	0.76
